# Predictive Value of High-Sensitivity CRP Level on the No-Reflow Phenomenon in STEMI Patients

**DOI:** 10.1155/crp/9359830

**Published:** 2025-06-30

**Authors:** Xhevdet Krasniqi, Josip Vincelj, Dardan Koçinaj, Blerim Berisha, Aurora Bakalli

**Affiliations:** ^1^Department of Internal Medicine, Medical Faculty, University of Prishtina “Hasan Prishtina”, Prishtina, Kosovo; ^2^Department of Cardiology, University Clinical Center of Kosovo, Prishtina, Kosovo; ^3^Department of Cardiovascular Medicine, Dubrava University Hospital, Zagreb, Croatia; ^4^Department of Internal Medicine, Kreisklinik Roth, Bavaria, Germany

**Keywords:** C-reactive protein, no-reflow phenomenon, STEMI

## Abstract

**Background:** Increased level of high-sensitivity C-reactive protein (hs-CRP) is associated with no-reflow phenomenon. Therefore, even when timely coronary revascularization is performed through the primary percutaneous coronary intervention (pPCI), the process can induce reperfusion injury.

**Purpose:** We evaluated the influence of hs-CRP level on the no-reflow phenomenon in patients with ST-segment elevation myocardial infarction (STEMI).

**Methods:** In this study, we included one hundred and eighty-two consecutive patients with STEMI of onset < 12 h, who underwent pPCI. The levels of creatine kinase (CK), the MB fraction of creatine kinase (CK-MB), troponin I, hs-CRP, and other routine laboratory parameters were measured. Measurement of hs-CRP was done on the day of admission by Cobas assay (particle-enhanced immunoturbidimetric assay) on Cobas c501 (Roche). Thereafter, patients were divided in two groups according to the thrombolysis in myocardial infarction (TIMI) flow grade.

**Results:** From a total of 182 STEMI patients who underwent pPCI, the median value of hs-CRP of the patients with TIMI grade flow 3 (successful reperfusion) was 8.5 (0.4–268) and of the patients with no-reflow phenomenon (unsuccessful reperfusion, TIMI flow grade ≤ 2) was 37.90 (1.8–271.20), *p* < 0.0001. Receiver operating characteristics (ROC) curve of hs-CRP plots the true positive rate against the false positive rate at different cutoff points, AUC = 0.73 (95% CI, 0.64–0.81), and the cutoff value for the hs-CRP was 18.0 mg/L, *p*=0.0001.

**Conclusions:** hs-CRP may be associated with no-reflow phenomenon in STEMI patients. The cutoff value for hs-CRP may be used to identify patients at risk for reperfusion injury.

## 1. Introduction

Ischemic heart disease (IHD) is the most common cause of death and disability worldwide.

Myocardial infarction (MI) is associated with cardiac troponin (cTn) release detecting the presence of acute myocardial injury [1]. Based on the initial electrocardiogram (ECG), patients with acute coronary syndrome (ACS) can be differentiated into two working diagnoses: ST-segment elevation MI (STEMI) and non-ST-elevation (NSTE)-ACS [2].

In patients with STEMI, even prompt reperfusion by primary percutaneous coronary intervention (pPCI) in-hospital mortality rate ranges between 4% and 12%, 1-year mortality is 7%, and goes up to 12% in-high risk STEMI patients that are presented with cardiogenic shock [3–5].

**Figure 1:**
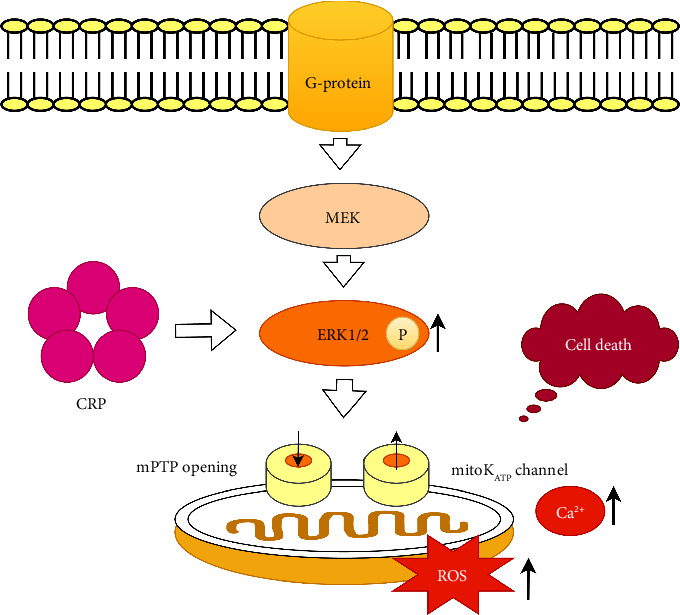
C-reactive protein and ERK pathway. CRP: C-reactive protein, ERK: extracellular signal-regulated kinase, MEK: mitogen extracellular kinase, ROS: reactive oxygen species.

The role of C-reactive protein (CRP) as predictor for inflammatory marker should be emphasized since it can clearly present the prediction on the no-reflow phenomenon. Thus, the CRP as a marker of acute inflammatory response may be comprised in myocardial ischemia/reperfusion injury in STEMI patients undergoing PCI (Figure 1) [6].

The CRP as marker of the elevated inflammatory state including albumin (the ratio of CRP to albumin-CAR) is used in predicting long-term mortality among heart failure with reduced ejection fraction (HFrEF) patients with implantable cardiac defibrillators as well as for the prediction of atrial fibrillation recurrence following cryoablation [7, 8]. Also, CRP can directly reflect the acute inflammatory responses to diseases [9].

The CRP gene is located on chromosome 1q23 while the CRP molecule itself consists of five nonglycosylated polypeptide subunits, where each contains 206 amino acid residues. The majority of CRP is synthesized in the liver but also in atherosclerotic lesions being regulated by interleukin-6 (IL-6), interleukin-1(IL-1), and tumor necrosis factor alpha (TNF-ɑ) [10, 11].

Reperfusion of an occluded coronary artery is required to prevent infarction of the ischemic myocardium, but reperfusion can trigger myocardial injury, a phenomenon called myocardial reperfusion injury (MRI). The pathogenesis of ischemia reperfusion injury consists of the mitochondrial permeability transition pore (mPTP) and the mitoK_ATP_ channel opening, generation of reactive oxygen species (ROS) and Ca^2+^ overload (Figure 1) [12].

The extracellular signal-regulated kinase 1/2 (ERK1/2) belongs to the mitogen-activated protein kinase (MAPK) family playing an essential role in signal transduction from surface receptors to the nucleus. CRP induces ERK 1/2 phosphorylation and is accompanied by ROS overproduction (Figure 1) [13].

The types of myocardial ischemia reperfusion injury include myocardial stunning, no-reflow phenomenon (microvascular damage), reperfusion arrhythmia, and lethal myocardial reperfusion injury. Based on the thrombolysis in myocardial infarction (TIMI) flow grade, no-reflow phenomenon is characterized by a TIMI flow ≤ 2 [14]Therefore, even when timely coronary revascularization is performed through the administration of thrombolytic therapy or a pPCI, the process can induce reperfusion injury. During this process, cardiomyocyte death occurs through several pathophysiological mechanisms and contributes up to 50% of the final myocardial infarct size [14].

Therefore, the prediction of no-reflow is very important in patients with STEMI. These laboratory markers such as CRP and ECG markers can be used in order to predict these endpoints [15]. Furthermore, artificial intelligence (AI) and machine learning (ML) systems can be used in order to precisely predict no-reflow [16].

To the best of our knowledge, no study has investigated the predictive value of high-sensitivity CRP level on the no-reflow phenomenon in STEMI patients.

The purpose of this study was to assess relationship between hs-CRP and TIMI flow grade as well as to determine the cutoff value for hs-CRP that may be used to identify patients at risk for no-reflow phenomenon.

## 2. Methods

### 2.1. Study Design

In this prospective observational study, we included one hundred and eighty-two consecutive patients with STEMI. These patients were enrolled in our center from March 2023 to February 2024.

Inclusion criteria for STEMI patients were (a) persistent chest pain, (b) an ECG with new ST elevation at the J-point in at least two contiguous leads (≥ 2.5 mm in men < 40 years, ≥ 2 mm in men ≥ 40 years, or ≥ 1.5 mm in women regardless of age in leads V2-V3 and/or ≥ 1 mm in the other leads (in the absence of left ventricular [LV] hypertrophy or left bundle branch block [LBBB])), (c) increased hs-cardiac troponin (hs-cTn) (ESC 0/1 h and 0/2 h algorithms), and (d) patients who underwent pPCI (persistent ST-segment elevation and symptoms of ischemia of ≤ 12 h duration) [2].

Clinical history data were obtained during hospitalization and included coronary risk factors (diabetes mellitus, dyslipidemia, arterial hypertension, and smoking), previous medications, and time from onset to admission.

Before PCI procedure, all patients received acetylsalicylic acid 150–300 mg, clopidogrel 600 mg, and unfractionated heparin (UFH) 70–100UI/kg. Additional treatment with glycoprotein IIb/IIIa inhibitors or intracoronary treatments such as vasodilators were left at the discretion of the treating cardiologist. Based on coronary angiography, no-reflow phenomenon was defined as TIMI flow grade ≤ 2 (unsuccessful reperfusion) [17, 18].

An echocardiographic examination was performed in all patients for assessment of left ventricular ejection fraction (EF).

Laboratory parameters were measured, including the creatine kinase (CK), the MB fraction of creatine kinase (CK-MB), hs-cTnI, hs-CRP, and other routine laboratory parameters were measured. Measurement of hs-CRP was done on the day of admission by Cobas assay (particle-enhanced immunoturbidimetric assay) on Cobas c501 (Roche).

The institutional ethic committee on human research approved this study.

### 2.2. Statistical Analysis

The main objective of the study was the influence of the hs-CRP on the no-reflow phenomenon in STEMI patients. The data follow a non-normal distribution, and nonparametric tests were taken into account in group comparisons. Mean ± standard deviation and median were used to present continuous variables while by count and percentage were expressed for categorical variables. Comparison of laboratory variables between patients with different TIMI flows (TIMI flow ≤ 2 or 3) was performed using Kruskal–Wallis and Mann–Whitney tests.

A receiver operating characteristic (ROC) curve as a graphical method plots the true positive rate against the false positive rate at different cutoff points, allowing the most appropriate cutoff to be chosen for the particular context. We used ROC curve analyses to determine a cutoff value for hs-CRP and its association with TIMI flow grade. The area under the curve is used as a summary measure of how well a variable predict a binary outcome. All statistical analyses were performed using SPSS statistics Version 22.

## 3. Results

We studied 182 STEMI patients admitted to the coronary care unit in the cardiology clinic. Baseline characteristics of the study population are presented in Table 1. The mean age was 63.17 ± 10.48 years old and 112 (61.53%) were male. Medical history included hypertension (57.69%), diabetes mellitus (24.17%), smoking (44.5%), and dyslipidemia (42.85%). The median value of the hs-CRP was 31.2 (0.40–271.20), while the median value of the CK-MB and troponin I were 90.00 (32.40–929.0) and 75.0 (25.0–6030.0). In terms of coronary angiographic findings, the culprit lesion was as follows: the left anterior descending artery (LAD) 41.2%, right coronary artery (RCA) 39.56% and left circumflex artery (LCx) 19.23%.

**Figure 2:**
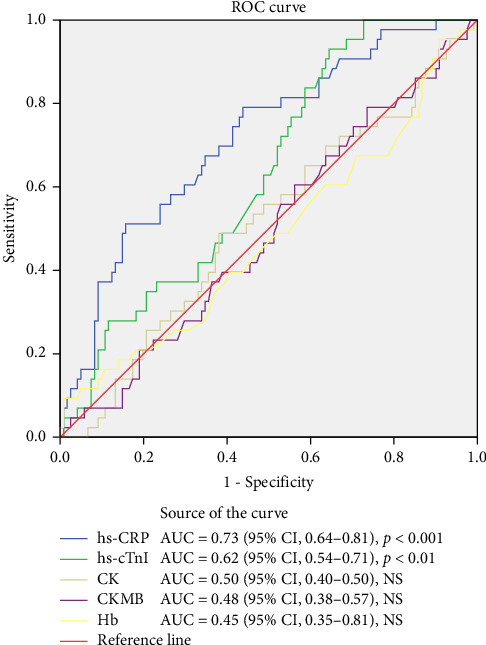
ROC curve analysis of biomarkers for the prediction of TIMI flow grade.

Of the 182 patients, 132 (72.52%) had TIMI flow grade 3 and 50 (27.47%) had TIMI flow grade ≤ 2. The mean age was 63.09 (±10.97) in patients with TIMI = 3 and 63.38 (±9.13) in those with TIMI ≤ 2. As for the laboratory parameters, the median value of hs-CRP of the patients with TIMI grade flow 3 was 8.5 (0.4–268) and 37.90 of those with no-reflow phenomenon (TIMI flow grade ≤ 2) (1.8–271.20) (*p* < 0.0001) (Table 2). ROC curve of hs-CRP plots the true positive rate against the false positive rate at different cutoff points, AUC = 0.73 (95% CI: 0.64–0.81), the cut off value for the hs-CRP was 18.0 mg/L, *p*=0.0001 (Figure 2). Also, Figure 2 presents the ROC curve analysis for others biomarkers (troponin I, CK, CK-MB, and hemoglobin).

## 4. Discussion

In this study, we investigated the impact (the predictive value) of hs-CRP level on the no-reflow phenomenon in STEMI patients.

Treatment of STEMI with percutaneous coronary intervention (PCI) as a preferable reperfusion therapy, despite opening of the occluded coronary artery, may be complicated with no-reflow phenomenon as strong evidence for myocardial reperfusion injury [19, 20].

CRP is an indicator of acute-phase inflammation and a mediator of atherosclerosis stimulating expression of adhesion molecules and inflammatory cells. It enhances uptake of oxidized low-density lipoprotein into macrophage through expression of tissue factor leading to plaque formation and thrombosis. CRP is also thought to contribute to vasoconstriction at the microvascular level and endothelial damage in which no-reflow is involved. Leukocytes play a key role in the pathophysiology of no-reflow as it accumulates in small vessel beds in the infarct zone. Aggregation of activated leukocytes in the capillary region may directly disrupt blood flow.

In addition to CRP, serum albumin (SA) levels can be used as biomarkers of systemic inflammation knowing its protective effects against endothelial damage and thrombotic state. Albumin is a negative acute-phase reactant and its serum concentrations are expected to decline in proinflammatory conditions such as atherosclerotic heart disease. C-reactive protein to serum albumin ratio (CRA) is used as a marker of cardiovascular disease. It has shown to be associated with no-reflow phenomenon and it is significant factor for coronary thrombus burden in STEMI patients undergoing PCI [21–24].

No-reflow is a dynamic and complex phenomenon as a result of alteration of coronary microcirculation where one of the main mechanisms is reperfusion injury [25].

Upon reperfusion injury, ROS were significantly induced following with endothelial dysfunction, deoxyribonucleic acid (DNA) damage and local inflammation causing cell death (Figure 1) [26, 27].

The influence of CRP on myocardial ischemia-reperfusion injury (MIRI) is mediated through extracellular regulated protein kinases 1/2 (ERK1/2) increasing production of ROS and Ca^2+^ [13, 28]. ERK1/2 is a serine/threonine protein kinase belonging to the mitogen-activated protein kinase (MAPK) family, which is abnormally expressed in MIRI (Figure 1) [29, 30]. In our study, ROC curve analysis of hs-CRP for the prediction of the no-reflow phenomenon (unsuccessful reperfusion, TIMI flow grade ≤ 2) was statistically significant, AUC = 0.73 (95% CI: 0.64–0.81), *p*=0.0001, confirming its influence as well as on MIRI (Figure 2).

Thus, in our study, the group of patients with no-reflow (unsuccessful reperfusion, TIMI flow grade ≤ 2) was characterized with higher value of hs-CRP compared with the group of patients with successful reperfusion (TIMI flow grade = 3), *p* < 0.0001 (Table 2). This study demonstrates that in patients with higher value of hs-CRP may be aggravated MIRI, as also investigated in preclinical studies [31].

Moreover, another result of this study shows that the level of cardiac troponin I (cTnI) can help to predict no-reflow phenomenon (*p*=0.01) as well as diabetes mellitus is associated with no-reflow (*p*=0.004) (Table 2) [32].

## 5. Conclusion

hs-CRP may be associated with no-reflow phenomenon in STEMI patients. The cut-off value for hs-CRP (18 mg/L) may be used to identify patients at risk for no-reflow phenomenon/reperfusion injury; therefore, additional treatment options should be kept in mind.

## Figures and Tables

**Table 1:** Baseline characteristics of patients.

Characteristics	Value
Age (yr)	63.17 (±10.48)
Gender (male), *n* (%)	112 (61.53)
BMI (kg/m^2^)	28.07 (±4.65)
Medical history	
Hypertension, *n* (%)	105 (57.69)
Diabetes mellitus, *n* (%)	44 (24.17)
Smoking, *n* (%)	81 (44.5)
Dyslipidemia, *n* (%)	78 (42.85)
Ejection fraction (%)	49.75 (±9.06)
Laboratory values	
Hemoglobin (mg/dL)	132.57 (±17.25)
Creatinine (μmol/L)	96.0 (43.0–479.9)
Creatine kinase (U/I)	907.0 (175.20–6809.0)
Creatine kinase-MB (U/I)	90.0 (32.40–929.0)
Troponin I (pg/mL)	75.0 (25.0–6030.0)
hs-CRP (mg/L)	31.2 (0.4–271.2)
Glucose (mmol/L)	10.82 (6.06)
Cholesterol (mmol/L)	5.08 (1.17)
Triglyceride (mmol/L)	2.07 (1.45)
Coronary angiographic findings	
Culprit lesion, *n* (%)	
LAD	75 (41.20)
LCx	35 (19.23)
RCA	72 (39.56)
Final TIMI grade flow, *n* (%)	
3	132 (72.52)
2	50 (27.47)

*Note:* Data are presented as a median (range), number of subjects (percentage), or mean ± SD.

Abbreviations: BMI, body mass index; TIMI, thrombolysis in myocardial infarction.

**Table 2:** Main characteristics and TIMI flow grade.

Characteristics	TIMI flow grade = 3 (*n* = 132)	TIMI flow grade ≤ 2 (*n* = 50)	*p* value
Age (yr)	63.09 (±10.97)	63.38 (±9.13)	0.85
BMI (kg/m^2^)	29.32 (4.46)	28.07 (4.65)	0.09
Hypertension, *n* (%)	75 (56.81)	30 (60.0)	0.23
Diabetes mellitus, *n* (%)	23 (17.42)	21 (42.0)	0.004
Smokers, *n* (%)	65 (49.24	16 (32.0)	0.12
Dyslipidemia, *n* (%)	52 (39.39)	26 (52.0)	0.65
Ejection fraction (%)	49.75 (±9.06)	45.79 (±9.46)	< 0.0001
Hemoglobin (mg/dL)	133.07 (±16.25)	131.2 (±19.61)	0.32
Creatinine (μmol/L)	96.2 (43.0–314.6)	93.20 (51.8–479.9)	0.34
Creatine kinase (U/I)	891.5 (175.20–7550.0)	1129.0 (180.0–6809.0)	0.9
Creatine kinase-MB (U/I)	93.0 (32.40–910.0)	140.51 (40.1–929.0)	0.65
hs-cTnI (pg/mL)	63.8 (25.0–5405.0)	86.07 (32.0–6030.0)	0.01
hs-CRP (mg/L)	8.5 (0.4–268)	37.90 (1.8–271.20)	< 0.0001
Glucose (mmol/L)	10.38 (6.03)	11.99 (6.05)	0.09
Cholesterol (mmoL/L)	5.11 (1.18)	5.0 (1.15)	0.58
Triglyceride (mmol/L)	2.13 (1.55)	1.91 (1.13)	0.82

## Data Availability

The data used to support the findings of this study are available on request from the corresponding author.
